# Geomicrobiology of sublacustrine thermal vents in Yellowstone Lake: geochemical controls on microbial community structure and function

**DOI:** 10.3389/fmicb.2015.01044

**Published:** 2015-10-26

**Authors:** William P. Inskeep, Zackary J. Jay, Richard E. Macur, Scott Clingenpeel, Aaron Tenney, David Lovalvo, Jacob P. Beam, Mark A. Kozubal, W. C. Shanks, Lisa A. Morgan, Jinjun Kan, Yuri Gorby, Shibu Yooseph, Kenneth Nealson

**Affiliations:** ^1^Thermal Biology Institute, Montana State UniversityBozeman, MT, USA; ^2^Land Resources and Environmental Sciences, Montana State UniversityBozeman, MT, USA; ^3^Center for Biofilm Engineering, Montana State UniversityBozeman, MT, USA; ^4^DOE Joint Genome InstituteWalnut Creek, CA, USA; ^5^J. Craig Venter InstituteLa Jolla, CA, USA; ^6^Eastern OceanicsWest Redding, CT, USA; ^7^US Geological SurveyDenver, CO, USA; ^8^Department of Earth Sciences, University of Southern CaliforniaLos Angeles, CA, USA

**Keywords:** metagenome, Aquificales, *Archaea*, hydrogen, sulfide, methane, thermophiles, methanotrophs

## Abstract

Yellowstone Lake (Yellowstone National Park, WY, USA) is a large high-altitude (2200 m), fresh-water lake, which straddles an extensive caldera and is the center of significant geothermal activity. The primary goal of this interdisciplinary study was to evaluate the microbial populations inhabiting thermal vent communities in Yellowstone Lake using 16S rRNA gene and random metagenome sequencing, and to determine how geochemical attributes of vent waters influence the distribution of specific microorganisms and their metabolic potential. Thermal vent waters and associated microbial biomass were sampled during two field seasons (2007–2008) using a remotely operated vehicle (ROV). Sublacustrine thermal vent waters (circa 50–90°C) contained elevated concentrations of numerous constituents associated with geothermal activity including dissolved hydrogen, sulfide, methane and carbon dioxide. Microorganisms associated with sulfur-rich filamentous “streamer” communities of Inflated Plain and West Thumb (pH range 5–6) were dominated by bacteria from the Aquificales, but also contained thermophilic archaea from the Crenarchaeota and Euryarchaeota. Novel groups of methanogens and members of the Korarchaeota were observed in vents from West Thumb and Elliot's Crater (pH 5–6). Conversely, metagenome sequence from Mary Bay vent sediments did not yield large assemblies, and contained diverse thermophilic and nonthermophilic bacterial relatives. Analysis of functional genes associated with the major vent populations indicated a direct linkage to high concentrations of carbon dioxide, reduced sulfur (sulfide and/or elemental S), hydrogen and methane in the deep thermal ecosystems. Our observations show that sublacustrine thermal vents in Yellowstone Lake support novel thermophilic communities, which contain microorganisms with functional attributes not found to date in terrestrial geothermal systems of YNP.

## Introduction

Submarine and sublacustrine thermal vents are found throughout the world and support an enormous diversity of life. Hydrothermal vent fluids often contain high concentrations of reduced constituents such as iron, sulfide, hydrogen, methane, arsenic, and/or ammonia that provide numerous possibilities for chemolithotrophic metabolism (Reysenbach et al., 2000; Amend and Shock, 2001; Coumou et al., 2008), as well as carbon dioxide important for supporting autotrophic organisms (Lovalvo et al., 2010). Hydrothermal discharge creates complex and dynamic temperature and geochemical gradients upon mixing with colder waters; the microorganisms that colonize different niches surrounding hydrothermal vents are of considerable interest in marine biology (e.g., Van Dover et al., 2001, 2007; Harmer et al., 2008), in part due to the potential microbial linkages with element cycling as well as the evolutionary implications of thermophilic organisms in marine settings (Reysenbach et al., 2000). The presence of eukaryotic mutualists adjacent to hydrothermal vents is often made possible by microbial symbionts capable of chemolithotrophic metabolism using reduced constituents present in vent fluids (Harmer et al., 2008; Setoguchi et al., 2014). Consequently, thermal vent microorganisms often conduct redox transformations and/or provide a source of nutrients important in the evolution of eukaryotes.

**Figure 1:**
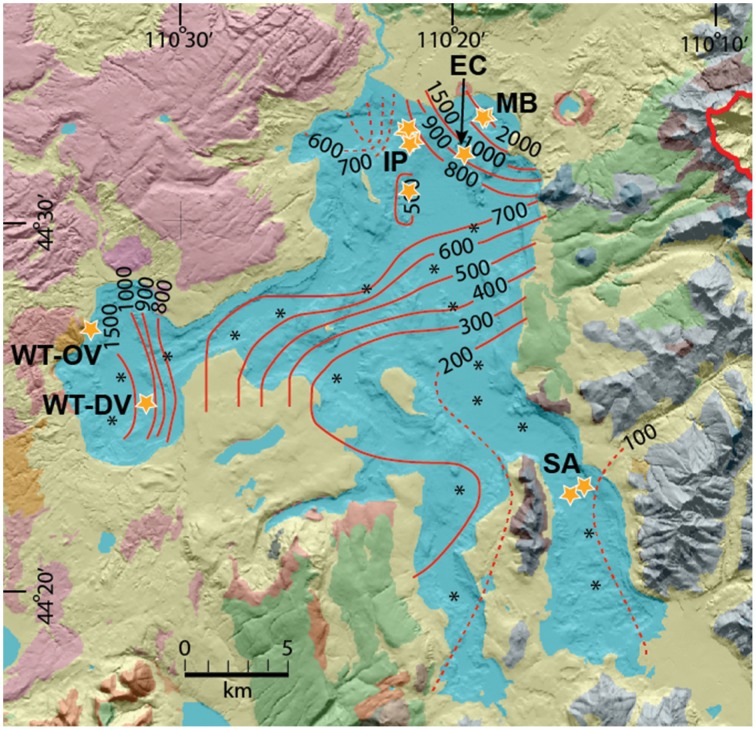
**Bathymetric map (Morgan and Shanks, 2005) of Yellowstone Lake showing heat flux iso-lines (mW/m^2^) (Morgan et al., 1977) and sampling locations of thermal vents (**Table 1**) discussed in the current study (IP, Inflated Plain; WT-DV, West Thumb Deep Vents; WT-OV, West Thumb Otter Vent; EC, Elliott's Crater; MB, Mary Bay; SA, Southeast Arm; see Table S1 for GPS coordinates)**.

Prior mapping and detailed geophysical analysis of Yellowstone Lake has provided critical information on the volcanology, geologic history and current location of major thermal activity on the lake floor (Morgan et al., 2003; Morgan and Shanks, 2005; Shanks et al., 2005). Prior sampling of hydrothermal vents in Yellowstone Lake provided important background information regarding the location and characteristics of different vent types (Johnson et al., 2003; Morgan et al., 2003, 2007; Morgan and Shanks, 2005; Shanks et al., 2005). The northern region of Yellowstone Lake is one of the most seismically active areas in Yellowstone Park and supports high geothermal heat fluxes of 500–2000 mW m^−2^ (Figure 1). Mary Bay itself was created as a result of an explosion crater that occurred approximately 0.2 Ma (Wold et al., 1977), and numerous other smaller features in this region attest to a dynamic and recent volcanic history (Morgan et al., 2009). The isotopic and geochemical composition of Yellowstone lake waters, vent waters and tributaries have shown that elevated levels of numerous trace elements (As, Se, B, Li, Cs, Ga) in Yellowstone Lake are due to hydrothermal inputs that represent ~10% of the total chloride flux from all of the geothermal features in YNP (Shanks et al., 2005, 2007; Balistrieri et al., 2007). Moreover, Cl^−^ vs. ^2^H_2_O plots place submerged vents in Yellowstone Lake on a mixing line between lake bottom-water and thermal fluids, which have an approximate temperature of 220°C (Shanks et al., 2005). High levels of trace elements, major nutrients, and/or energy sources near vent discharge have been shown to influence the diversity and productivity of biological communities in Yellowstone Lake (Lovalvo et al., 2010; Clingenpeel et al., 2011, 2013; Kan et al., 2011; Yang et al., 2011).

Efforts to characterize microbial communities from several vent sites in Yellowstone Lake using modest bacterial 16S rRNA gene surveys have shown that thermophilic bacteria from the order Aquificales were important in sulfidic habitats (Yang et al., 2011). Sulfur oxidizing Proteobacteria were also important in several vent sites, including organisms related to *Thiovirga* spp., *Thiobacillus* spp., and *Sulfuricurvum* spp. Geochemical analyses of the higher-temperature (i.e., >50°C), deeper (>49 m) vent sites (3) confirmed high levels of sulfide and other reduced sulfur species, which upon mixing with oxygenated lake water, provide habitats suitable for sulfur-oxidizing microbial communities, and which support significant rates of dark CO_2_ fixation (Yang et al., 2011).

The prior geochemical work on Yellowstone Lake thermal vents (Shanks et al., 2005; Balistrieri et al., 2007), as well as efforts to characterize microorganisms present in these communities (Yang et al., 2011), or in filtered vent fluids (Clingenpeel et al., 2011, 2013; Kan et al., 2011), suggested that thermal vents in Yellowstone Lake contain thermophilic communities whose functional attributes can be correlated with pronounced chemosynthetic gradients. Moreover, several sublacustrine vents in Yellowstone Lake exhibit unique chemical signatures that support novel assemblages of both *Bacteria* and *Archaea*. Here we report an integrated study of hydrothermal vent geochemistry, and associated molecular and microscopic analysis of microbial communities from several of the major vent types in Yellowstone Lake (YNP, USA). The primary objectives of the study were to (i) determine the geochemical composition of hydrothermal vent fluids and predominant solid phases associated with hydrothermal vents in Yellowstone Lake, (ii) identify predominant thermophilic microbial populations inhabiting major vent types in Yellowstone Lake using both 16S rRNA gene and random shotgun sequencing, and (iii) compare differences in functional genes observed in metagenome sequence obtained from vents exhibiting different geochemical signatures. Geochemical analysis indicated that thermal vents in Yellowstone Lake contain high concentrations of dissolved gases including H_2_S, H_2_, CH_4_, and CO_2_, as well as various trace elements and hydrogen ions (pH values ranged from 5 to 6.4 in deep vents, compared to bulk lake water pH = 7.0). Our results showed a definitive linkage between vent chemistry, microbial community structure, and associated metabolic attributes of microorganisms supported by high-temperature systems in Yellowstone Lake.

## Results and discussion

### Geochemical analysis of sublacustrine thermal vents in YNP

#### Aqueous samples

Temperature values measured at the sampling end of the suction arm (Table 1) confirmed that all vent waters collected with the ROV (Figure S1) had received significant inputs of hydrothermal water, and/or had been heated due to adjacent thermal activity. The large range in vent temperature(s) at a single sampling location was due to the dynamics of mixing with surrounding lake water at temperatures of 8–10°C. In most cases, stable temperatures above 60°C were maintained for extended measurement periods of 1–2 h during fluid collection. The concentrations of many constituents considered signatures of geothermal activity, such as dissolved CO_2_, H_2_, H_2_S, and CH_4_ were considerably higher in thermal vent waters relative to background lake water (e.g., Southeast Arm, Table 1). The deep thermal vents were all mildly acidic compared to bulk lake water, ranging from pH 5.1 at Mary Bay (MB), 5.2–5.6 at Inflated Plain (IP), 5.9–6.2 at West Thumb (WT), and 6.2–6.4 at Elliot's Crater (EC). A shallow (4.3 m) “alkaline siliceous” thermal vent on the west side of WT (i.e., the Otter Vent) exhibited a pH ~ 8.2. Lower pH values at MB and IP were correlated with higher concentrations of Fe and Al (Table S1), consistent with mineral solubility as a function of pH. Other key indicator constituents of geothermal inputs were observed at concentrations significantly higher than background lake water (>5–10x), and included F, NH_4_, As, Sb, W, Mo, Li, Cs, B, and/or Na (Table S1). Concentrations of major cations (Ca, Mg, K) and anions (Cl, SO_4_) were generally similar in vent vs. lake waters, although vent waters at WT revealed high levels of Cl and SO_4_, as well as Na.

**Table 1:** **Key geochemical characteristics^a^, temperature values and sample depths of sublacustrine thermal vent waters (and lake water from the Southeast Arm) obtained from Yellowstone Lake using the remotely operated vehicle (ROV) during September 2007 and 2008**.

**Sample**	**Depth**	**Temp**	**pH**	**DIC^a^ CO_2_ (aq)^b^**		**DS^a^**	**O_2_(aq)^b^**	**CH_4_ (aq)^b^**	**H_2_ (aq)^b^**	**Sample**	
**Location**	**m**	**°C**		**mM**		**μM**			**nM**	**ID^c^**	**Date**
Inflated Plain	30	92–94	5.6	8.5	8.1	633	bd	21.8	414	329-Sy-1	9/9/2007
	32	70–76	5.6	4.1	3.2	463	25	21.2	4837	330-Sy-1	9/10/2007
	32	40–45	5.2	3.1	3.1	230	bd	20.9	1031	348-Sy-P	9/11/2008
	30	40–60	5.2	3.2	3.2	266	bd	22.5	1430	348-Sy-S	9/11/2008
	33.6	44–52	5.5	1.2	1.2	85	25	6.7	1023	359-VC	9/16/2008
	33.6	41–49	5.7	1.1	1.1	111	bd	5.4	1974	359-Sy-S	9/16/2008
West Thumb	52	60–66	6.2	4.7	1.6	2	113	6.4	41	339-VC	9/18/2007
Deep Vent	52	60–66	6.2	nd^d^	1.8	bd^e^	bd	7.2	63	339-Sy	9/18/2007
	52	60-76	6.2	1.3	0.4	1	bd	14.5	30	341-Sy-1	9/19/2007
	54	66	6.1	2.5	1.0	8	188	4.6	102	343-Sy-1	9/19/2007
	53.2	40-53	6.1	nd	2.1	10	147	5.6	23	369-VC	9/20/2008
	53.2	38	5.9	nd	2.0	10	238	6.8	33	369-Sy-P	9/20/2008
	53.2	62-66	5.9	nd	3.0	13	210	10.5	41	369-Sy-S	9/20/2008
Mary Bay	49.6	30-57	5.1	4.0	3.3	172	bd	13.7	454	335-Sy-S	9/16/2007
	52.3	62-66	5.1	6.4	4.5	385	bd	17.9	511	336-Sy-1	9/16/2007
	52.3	73-74	5.1	5.9	6.0	433	bd	20.4	100	336-Sy-2	9/16/2007
	50.5	65-70	5.0	3.8	3.8	bd	bd	28.1	2984	349-Sy-P	9/12/2008
	50.5	62-70	5.4	1.8	1.8	123	94	12.4	2797	349-Sy-S	9/12/2008
Elliot's Crater	14.1	71	6.4	1.4	0.5	43	202	4.4	688	351-Sy-S	9/13/2008
	14.1	71	6.2	1.6	0.7	54	190	1.9	672	351-Sy-P	9/13/2008
	14.1	63-68	6.4	1.3	0.5	40	120	2.3	660	352-VC	9/14/2008
West Thumb	4.3	65-70	8.4	0.8	0.0	bd	47	2.1	551	332-Sy	9/11/2007
Otter Vent	4.3	63-68	8.4	0.7	0.0	bd	26	0.1	43	333-VC	9/12/2007
Southeast Arm	2.5	10.6	7.0	0.6	0.013	bd	234	0.1	10	344	9/20/2007
	3	11	7.0	0.6	0.014	bd	313	0.2	47	354	9/15/2008
	17	10.5	7.1	0.6	0.019	bd	344	0.1	23	356	9/15/2008

a DIC, dissolved inorganic C; DS, dissolved sulfide; other constituents given in Table S1.

b Dissolved gas species determined using headspace GC, aq, aqueous.

c Sy, ROV syringe, P, port side, S, starboard side, VC, vent carboy/peristaltic pump.

d nd, not determined.

e bd, below detection; detection limit DS = 0.3 μM; O_2_ = 3 μM.

Dissolved gas [H_2_S(aq), CO_2_(aq), H_2_(aq), and CH_4_(aq)] concentrations from thermal vents were one to two orders of magnitude higher than in background lake water (Table 1), and were considerably higher than measured in terrestrial sites of YNP (Spear et al., 2005; Inskeep et al., 2013a). Vent waters from MB and IP contained the highest levels of total dissolved sulfide (DS), H_2_(aq), and CH_4_(aq), and were also the most acidic waters found in the study. Although, the concentrations of dissolved gases varied across different sample types collected for a given vent, the measurements were reasonably stable considering the sampling challenges presented under these circumstances (i.e., rapid mixing with bulk lake water). The large flux of H_2_S(g) from the IP vent region resulted in concentrations of DS well-above detection (e.g., 3–5 μM) in several surface (0–10 cm) lake samples obtained within discharge zones at IP.

#### Microscopy and solid phase analysis

**Figure 2:**
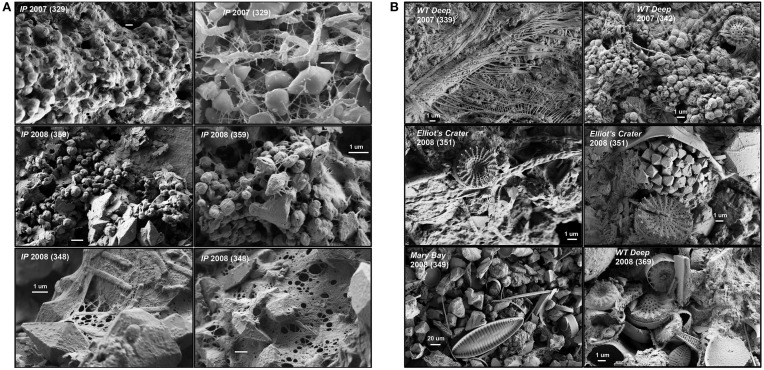
**(A)** Scanning electron micrographs of thermal streamer communities obtained from 30 to 33 m vents in the Inflated Plain region, Yellowstone Lake (Sample ID). All scale bars = 1 μm. **(B)** Scanning electron micrographs of thermal vent biomass samples obtained from vent sites at West Thumb deep (Sample ID 339, 342; 2007), Elliot's Crater (351; 2008), and Mary Bay (349; 2008). Sediments associated with thermal vents show accumulation of diatom shells (e.g., Mary Bay, 349, lower right), which were also trapped in filamentous streamer communities (e.g., West Thumb, 369, lower left).

Scanning electron microscopy (FE-SEM) of vent biomass provided considerable insight regarding the characteristics of each sample, and the potential processes responsible for the formation of filamentous structures. Images of the sulfur-rich streamers from IP (Figure S2) reveal coccoid, rod-shaped, and filamentous organisms contained in a complex extracellular matrix including rhombohedral crystals of elemental S (Figure 2A). Extracellular substances were a dominant feature observed in streamers from IP, and although the exact composition of these materials is not known, the resultant “streamer structures” are very resistant to dispersion and/or disaggregation. West Thumb streamers were notably more complex, and contained diverse cellular structures, less elemental S, and more diatom shells. The vent sediments collected from MB and EC also contained numerous diatom shells intermixed with a complex suite of siliceous minerals, aluminosilicates and organic material (Figure 2B). The extracellular matrix evident in the thermophilic IP streamers envelopes bundles of individual filaments and sulfur crystals into dense “liquid-like” structures that exhibit significant cohesion (Figure S2).

### Microbial community structure and function

**Figure 3:**
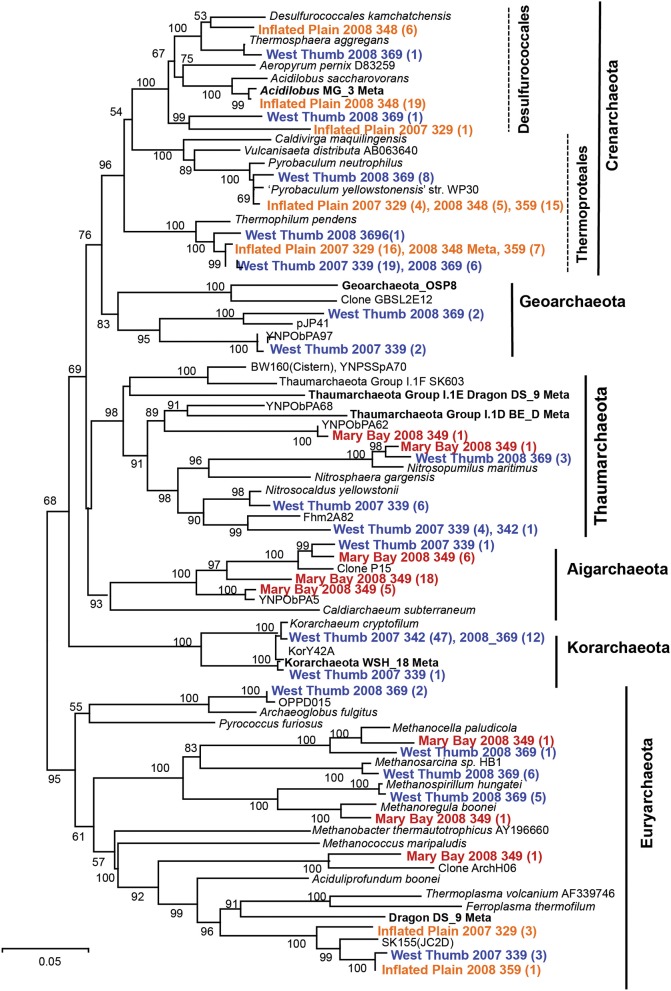
**Phylogenetic tree (16S rRNA gene sequences) of the domain *Archaea* including long-fragment sequences observed in thermal vent microbial communities from Yellowstone Lake (neighbor-joining tree; bootstrap values reported based on 1000reps Log Det.)**. All long-fragment 16S rRNA gene sequences from Yellowstone Lake are deposited in GenBank (KT453543-KT453636).

Long-fragment (>1000 bp) archaeal and bacterial 16S rRNA gene sequences indicated the major types of thermophilic microorganisms present in vent biomass (Table 2, Figure 3). *Sulfurihydrogenibium* spp. (order Aquificales) were a significant fraction of the bacterial populations observed in sulfur streamers from IP and WT, and these organisms are also found in sulfidic geothermal springs of YNP (Nakagawa et al., 2005; Reysenbach et al., 2005; Inskeep et al., 2010; Takacs-Vesbach et al., 2013). Other bacteria observed in streamer communities from IP and WT included *Caldisericum* (Candidate Division OP5), *Geothermobacterium, Sulfuricurvum, Thiovirga*, and *Thiobacillus* spp. (Proteobacteria), all of which are often found in sulfidic environments (Inskeep et al., 2005; Ito et al., 2005; Mori et al., 2009; Han et al., 2012). Deep (~50 m) vents at WT were the only samples to exhibit relatives of *Methylothermus thermalis* (Methylococcales), and these sequences comprised ~10, 28, and 64% of the bacteria observed in 3 independent vents from this region (Table 2).

**Table 2:** **Summary of predominant long-fragment bacterial 16S rRNA gene sequences (nt > 1200 bp) obtained from thermal vent biomass samples from Yellowstone Lake using clone-library analysis (*n* ~ 48 per site)**.

**Location year (ID)**	**Closest cultivated relative^a^**	**Taxonomic group^b^**	**Percent library**	**Clone ID**	**IDY (%)**	**Relative NCBI No**.
**Inflated Plain**	*Sulfurihydrogenibiumsp.* Y04ACS1	Aquificales	83	329-10-20	~99	AM259493.1
	*Geothermobacterium ferrireducens*	Thermodesulfobacteria	5	329-5	98.6	AF411013.1
2007 (329S)	*Caldisericum exile* AZM16c01	Candid. Div. OP5	5	329-21	98.3	NR_075015.1
	*Thiovirga sulfuroxydans*	Chromatiales	5	329-22	97.3	NR_040986.1
2008 (348S)	*Sulfurihydrogenibium* sp. Y04ACS1	Aquificales	100	348-5	>99	AM259493.1
2008 (359S)	*Sulfurihydrogenibium* sp. Y04ACS1	Aquificales	100	359-5	>99	AM259493.1
**West Thumb**	*Sulfurihydrogenibium yellowstonense*	Aquificales	30	339-15,16	>99	JQ346738.1
	*Thiovirga sulfuroxydans*	Chromatiales	20	339-20, 24	97.3	NR_040986.1
2007 (339)	*Methylothermus thermalis* MYH	Methylococcales	10	339-13	92.8	NR_043209.1
	*Caldisericum exile* AZM16c01	Candid. Div. OP5	10	339-17	98.5	NR_075015.1
2007 (342)	*Methylothermus thermalis* MYH	Methylococcales	64	342-4	93.0	NR_043209.1
	*Sulfurihydrogenibium* sp. Y03AOP1	Aquificales	10	342-12	99.4	NR_074557.1
	*Thiobacillus denitrificans*	Hydrogenophilales	10	342-5	91.0	NR_074417.1
	*Curvibacter* sp. PL21	Burkholderiales	3	342-7	98.9	KF206393.1
	*Thermus aquaticus*	Thermales	3	342-9	95.4	NR_025900.1
	*Bellilinea* sp. clone 96	Nov. Chloroflexi	3	342-8	< 95	JQ183076.1
2008 (369S)	*Methylothermus thermalis* MYH	Methylococcales	28	369-20	92.8	NR_043209.1
	*Sulfurihydrogenibium sp.* Y04ACS1	Aquificales	25	369-21	99.6	AM259493.1
	*Thiobacillus dentrificans*	Hydrogenophilales	6	369-25	96.1	NR_074417.1
	*Geothermobacterium ferrireducens*	Thermodesulfobacteria	3	369-19	98.3	AF411013.1
	*Fervidobacterium* sp. CBS-3	Thermotogales	3	369-18	91.3	EF222230.1
	*Syntrophomonas palmitatica MPA*	Syntrophobacterales	3	369-22	94.5	NR_041528.1
	*Thermodesulforhabdus norvegica*	Syntrophobacterales	3	369-23	91.8	NR_025970.1
	*Rhodoferax ferrireducens*	Burkholderiales	3	369-27	97.5	NR_074760.1
	*Thermus aquaticus*	Thermales	3	369-24	98.0	NR_025900.1
	*Desulfomicrobium thermophilum* P6.2	Uncl. Proteobacteria	3	369-16	96.8	NR_042924.1
	*Pelosinus* sp. UFO1	Firmicutes	3	369-268	94.0	DQ295866.1
**Mary Bay**	*Caldisericum exile* AZM16c01	Candid. Div. OP5	12	349-20	89.9	NR_075015.1
	*Thermanaerothrix daxensis* GNS-1	Chloroflexi	12	349-10	92.9	HM596746.1
2008 (349S)	*Cystobacter violaceus* Cbvi34	Myxococcales	12	349-11	93.2	KF267724.1
	*Desulfosarcina cetonica*	Desulfurobacteriales	12	349-12	92.1	NR_028896.1
	*Ornatilinea apprima*	Chloroflexi	8	349-15	88.9	NR_109544.1
	*Prosthecobacter fluviatilis*	Verrucomicrobiales	8	349-21	83.5	NR_041608.1
	*Methylobacter psychrophilus* Z-0021	Methylococcales	8	349-17	98.5	NR_025016.1
	*Geobacter daltonii*	Desulfomonadales	4	349-13	84.4	NR_074916.1
	*Syntrophus aciditrophicus SB*	Syntrophobacterales	4	349-19	83.2	NR_102776.1
	*Aquihabitans daechungensis*	Acidimicrobiales	4	349-14	93.5	NR_132289.1
**Elliot's Crater**	*Thiobacillus thioparus* DSM505	Hydrogenophilales	57	351-8	96.8	NR_117864.1
	*Bellilinea caldifistulae*	Chloroflexi	14	351-1	90.4	NR_041354.1
2008 (351)	*Sulfurihydrogenibium* sp. Y04ACS1	Aquificales	9	351-5	98.9	AM259493.1
	*Pelobacter massiliensis* DSM6233TN	Desulfuromonadales	4	351-4	85.3	FR749901.1
	*Denitratisoma sp.* TSA61	Rhodocyclales	4	351-132	94.9	AB542411.1
	*Thermomonas hydrothermalis* SGM-6	Xanthomonadales	2	351-7	98.0	NR 025265.1
	*Caldisericum exile* AZM16c01	Candid. Division OP5	2	351-130	97.4	NR 075015.1
**Otter Vent**	*Fervidobacterium changbaicum*	Thermotogales	27	332-7	94.7	NR 043248.1
	*Synechococcus* sp. TS-97 B'	Cyanobacteria	11	332-16	99.7	AY884056.1
2007 (332)	*Anaeromyxobacter* sp. K	Myxococcales	11	332-13	86.0	NR 074969.1
	*Thermodesulfoba. hydrogeniphilum*	Thermodesulfobacteria	11	332-17	79.6	NR 025146.1
	*Chloroflexus* sp. strain 396-1	Chloroflexi	8	332-5	98.1	AJ308498.1
	*Thiobacter subterraneus* C55	Burkholderiales	8	332-18	92.1	NR 024834.1
	*Acidobacteria bacterium* KBS 96	Acidobacteriales	5	332-3	91.8	FJ870384.1
	*Fischerella* sp. JSC-11	Cyanobacteria	5	332-12	99.9	HM636645.1
	*Anaerolinea thermophila* UNI-1	Anaerolineales	3	332-4	98.6	AP012029.1

a In some cases, clones are listed due to distant cultivated relatives. All Yellowstone Lake 16S rRNA gene sequences are deposited in GenBank [Accession Numbers KT453543 - KT453636].

b Major phylum, order, or family.

The sulfur streamers from WT contained 16S rRNA gene sequences representing 6 major lineages in the *Archaea* (Figure 3), including members of the Korarchaeota and Euryarchaeota, which were notably absent in replicate (temporal and spatial) streamer samples from IP. Archaea present in the sulfur streamers from IP were dominated by members of the Crenarchaeota (including the Desulfurococcales and Thermoproteales), as well as a novel group of Euryarchaeota (related to the Thermoplasmatales), which are also observed in sulfur sediments of terrestrial YNP springs (Inskeep et al., 2013b). The MB sediments also contained undescribed archaeal populations including members of the Aigarchaeota, Thaumarchaeota, and Euryarchaeota (primarily relatives of methanogens), although no Crenarchaeota were observed.

Compared to IP streamers, larger contributions of non-thermophilic bacteria were detected in samples from WT, MB, and EC. Bacterial sequences from MB sediments revealed an extensive diversity of different Proteobacteria, many of which are more closely related to moderate thermophiles and/or mesophiles often found in extreme sulfur and/or iron-rich habitats (e.g., Ito et al., 2005). The greater number of different bacterial sequence types observed in MB and EC sediments (Table 2) was consistent with sampling constraints at these locations, which resulted in collection of a significant amount of sediment adjacent to the vent exit walls. Bacterial sequences from the shallow phototrophic communities (pH 8.2) at the WT-Otter Vent (OV) corresponded to two major cyanobacterial groups (*Synechococccus* and *Fisherella* spp.), different members of the Chloroflexi, as well as major contributions (~27% of the clone library) from a novel Thermotogales population (*Fervidobacterium* spp.) (Table 2).

#### Pyro-tag sequencing

**Figure 4:**
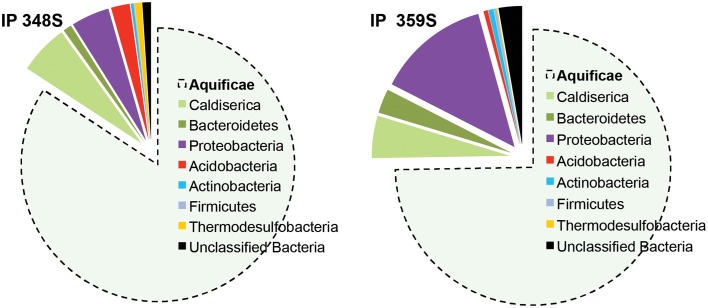
**Phylogenetic classification of short fragment bacterial 16S rRNA gene sequences from two different sulfur streamer communities from Inflated Plain (depth ~ 30–33 m; pH ~ 5.6) obtained using pyro-tag sequencing (sequences classified using RDP Naïve Bayesian rRNA Classifier version 2.5; also see Table 3)**.

Four vent biomass samples were subjected to more intensive 16S rRNA gene sequencing as well as random shotgun sequencing. The majority of phylotypes observed using pyro-tag sequencing (Table 3) of IP streamers (2 sites), WT streamers, and MB sediments were also found using long-fragment sequence analysis, and provided corroborative evidence of the major taxonomic groups present. Aquificales-like sequences (i.e., *Sulfurihydrogenibium* sp.) dominated the bacterial 16S rRNA gene libraries (74–84%) obtained from two IP sulfur streamers (Figure 4). Conversely, the WT streamers exhibited significantly greater bacterial diversity and contained only 10% Aquificales (Table 3), which is consistent with lower DS and H_2_(aq) relative to the vents at IP (Table 1). Mary Bay vent sediments contained very few Aquificales sequences, consistent with the lack of any notable streamers at this site, and the significant contribution from mesophilic organisms. Populations related to *Caldisericum exile* (Mori et al., 2009; candidate phylum OP5) were observed in all samples, but especially in association with the sulfur streamers at IP (Figure 4).

**Table 3:** **Major taxonomic groups (fraction of total bacterial or archaeal sequences) in vent-associated microbial communities determined using pyro-tag 16S rRNA gene sequencing of amplicons generated with universal bacterial (top) and archaeal (bottom) primer sets (Clingenpeel et al., 2011, 2013; Kan et al., 2011)**.

**Taxonomic groups^a^ Bacteria**	**Site name**			
	**IP 348S**	**IP 359S**	**WT 369S**	**MB 349S**
Aquificae	83.8	73.9	10.3	0.4
Caldiserica	5.8	4.9	0.7	2.6
Bacteroidetes	1.0	2.8	12.7	23.9
Proteobacteria	4.5	13.1	25.3	13.6
Acidobacteria	2.2	0.5	1.4	3.6
Chloroflexi	0.1	0.0	6.4	7.4
Thermotogae	0.0	0.1	7.1	0.1
Actinobacteria	0.3	0.6	1.3	5.0
Deinococcus-Thermus	0.0	0.0	4.5	0.0
Firmicutes	0.1	0.3	3.3	2.1
Cyanobacteria	0.1	0.0	1.0	12.7
Thermodesulfobacteria	0.8	0.1	2.4	0.1
Chlorobi	0.0	0.0	2.2	1.1
Planctomycetes	0.0	0.1	1.3	1.1
Gemmatimonadetes	0.0	0.0	0.5	1.6
Nitrospira	0.0	0.0	1.0	0.3
Unclassified Bacteria	1.0	2.7	15.6	21.5
Total^b^	99.7	99.1	97	97.1
n	27540	32471	27338	25753
**Archaea**	**IP**	**IP**	**WT**	**MB**
**CRENARCHAEOTA**				
Desulfurococcales	48.8	12.5	17.4	17.0
Thermoproteales	49.2	86.2	21.9	14.2
Sulfolobales	0.2	0.2	0.0	0.1
Other “Crenarchaeota”	1.8	0.9	2.4	22.8
**EURYARCHAEOTA**				
Methanomicrobiales	0.0	0.0	0.3	0.6
Thermoplasmatales	0.0	0.0	0.5	0.9
Novel Euryarchaeota	0.0	0.1	41.6	12.2
Korarchaeota	0.0	0.0	9.7	23.1
Unclassified Archaea	0.0	0.0	5.4	8.7
Total^b^	100.0	99.9	99.2	99.6
n	24191	13945	13495	26382

a RDP training set 9, RDP Naive Bayesian rRNA Classifier version 2.5, May 2012 Classifications performed March 18, 2013. Novel Crenarchaeota include what was referred to as “Marine Crenarchaeota,” now established within the Candidate phylum Thaumarchaeota.

b Total = percent of total sequences (n).

Other major groups of *Bacteria* varied with vent sites, but included members of the Bacteroidetes, Proteobacteria (the Epsilon group was more important in IP whereas Beta and Delta groups were more important in WT and MB), Thermotogae and Deinococcus-Thermus (7.2 and 4.5% in WT streamers), Acidobacteria (4% in *MB* sediments), Actinobacteria (5.4% in MB sediments), Thermodesulfobacteria (2.4% in WT), Planctomycetes ~2% in WT and MB), as well as members of the Chloroflexi (~6–8% in WT and MB sediments) (Table 3). It is unlikely that Chloroflexi-like sequences are contributed from organisms conducting photosynthesis at these depths; phylogenetic placement of long-fragment 16S rRNA sequences that were highly related to the shorter pyro-tag reads suggest that many of the Chloroflexi sequences were contributed by relatives of anaerobic, heterotrophic strains (Yamada et al., 2007; Klatt et al., 2013) (Table 3).

Different types of *Archaea* were observed across sites (Table 3), and the major groups identified using pyro-tag sequencing were also observed in long-fragment clone libraries (e.g., Table 2, Figure 3). The highly sulfidic and H_2_-rich IP streamers (pH ~ 5.2–5.6) exhibited a consistent signature of Crenarchaeota (>99% of archaeal reads), including members of the Thermoproteales (*Pyrobaculum* and *Thermofilum*-like populations) and Desulfurococcales (*Desulfurococcus* and *Acidilobus*-like sequences; Jay et al., 2014). Very few Sulfolobales sequences were observed, which is expected given the pH range of these vent communities (pH 5–6) (Macur et al., 2013; Jay et al., 2014). Members of the Korarchaeota were found primarily in the less sulfidic and higher pH streamers from WT, as well as in sediments from MB (Table 3). A significant number of novel euryarchaeotal sequences were observed in WT and MB, and represent several novel methanogens, an undescribed group related to the order Thermoplasmatales (~85% nt identity, Figure 3), as well as members of the Thaumarchaeota and Aigarchaeota (Brochier-Armanet et al., 2008; Nunoura et al., 2011). Long-fragment clone libraries also indicated the presence of different types of Euryarchaeota and Thaumarchaeota in WT streamers and MB sediments (Figure 3), including relatives of both low-temperature thaumarchaea (Hatzenpichler, 2012) as well as thermophilic clades (Beam et al., 2014). The korarchaeotal sequences observed using pyro-tag analysis (~10–23% of WT and MB pyro-tag sequences) corresponded to long-fragment 16S rRNA gene sequences, which were observed at several WT vent sites in both 2007 and 2008 (Figure 3).

#### Metagenome sequence analysis

**Figure 5:**
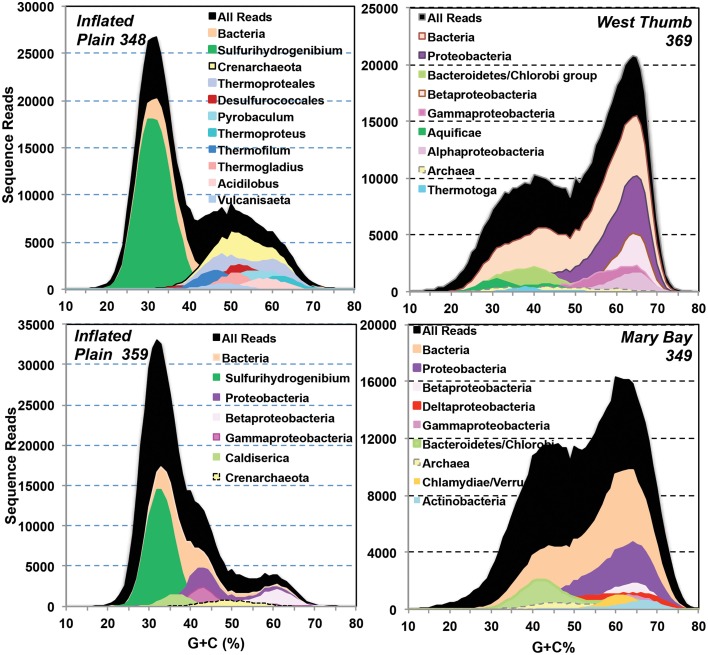
**Random shotgun sequence reads from four Yellowstone Lake thermal vent microbial communities plotted as a function of G + C content (%) and subjected to phylogenetic analysis using blast (90% identity)**. A significant number of sequence reads were not related to bacteria or archaea in current public databases.

Random shotgun sequence (average read length ~400 bp) obtained from four vent sites (IP, WT, and MB) was analyzed using Blastx (NCBI) and G + C content (%) to examine the predominant populations present in each site (Figure 5). The random sequence data indicated a lower abundance of archaea relative to bacteria in all vents sampled, representing from less than 5% of the total sequences in three of the four vent sites up to nearly 30% in one of the sulfur streamers from IP (348S). The major phylotypes identified with random sequence were also consistent with those observed using amplification techniques. For example, random sequence reads from two different streamer communities from IP were dominated by sequences related to *Sulfurihydrogenibium, Caldisericum*, and other Proteobacteria (Figure 5). The streamer communities from WT were dominated by sequences related to members of the Bacteroidetes, Aquificales, and Proteobacteria, and the sediments from MB contained a diverse assemblage of distant relatives of the Bacteroidetes (lower G + C), Proteobacteria (higher G + C), Chlamydiae/Verucomicrobia, and Actinobacteria. Much of the random shotgun sequence from MB (and to a lesser extent in WT) was not sufficiently similar to reference organisms (NCBI) to assign individual sequence reads to specific genera.

The amount of assembled genome sequence (Table S2) obtained from the four vent sites was inversely correlated with the number of dominant sequence types observed using 16S rRNA gene inventories. For example, the higher percent of reads assembled from IP streamers (348S and 359S) resulted in larger contigs with higher sequence coverage (Table S2). Phylogenetic assignment of 16S rRNA genes obtained from assembled sequence (Table S3) was consistent with populations observed using 16S rRNA gene-only approaches (Tables 2, 3, Figures 3, 4). Consequently, the sequence assemblies from IP and WT represent an excellent opportunity for linking specific metabolic genes with known phylotypes.

#### Functional gene analysis

The predominant energy cycling reactions mediated by microorganisms present in vent communities was investigated using specific query (marker) genes that code for proteins known to mediate the assimilation of inorganic C, electron transfer, and/or stress response (Table 4). Nearly, all phylotypes identified using different functional genes were consistent with those determined using phylogenetic analysis of 16S rRNA genes (e.g., Figures 3, 4, Tables 2, 3). Consequently, a consistent picture emerges regarding the functional attributes of major population types identified in IP and WT (Table 4). The low fraction of assembled sequence obtained from the MB vent sediments precluded confident assignment, and the majority of genes identified were less than 25–30% of their full length (not shown).

**Table 4:** **Summary of functional genes^a^ (and their phylogenetic identity^b^) related to key geochemical processes, which were identified in assembled metagenome sequence of three thermal vent microbial communities from Yellowstone Lake, WY**.

**Process/Pathway**	**Marker genes^a^**	**Inflated Plain (IP-348S)**	**Inflated Plain (IP-359S)**	**West Thumb (WT-369S)**
**FIXATION Of CO_2_**				
ATP citrate lyase	aclB	Sk, Sy	Sk, Sy	
Citryl coA lyase	ccl	Sy	Sy	Sy
Citryl coA synthetase	ccsA	Tu	Ce, Sk, Sy	At, Sy, Td
Acetyl-coA carboxylase	accA	Ce, Sy, Tp	Acid, Ce, Sk, Sy, Thio	Ma, Td
**OXIDATION REDUCTION**				
Hydrogen oxidation	hynS, hynL	Sy, Tp, Tu,	Sy, Tp	Td
Elemental S oxidation	hdrA/hdrB	Sy	Sy	Sy, Td
Sulfide oxidation	sqr	Sy,	Sk, Sy, Thio	Td,
Sulfur transferase	rdh	Sk, Sy	Sy, Sk	Nl, Sy, Td
Sulfur oxidation	soxBCDY	Sy,	Ce, Sy, Thio	Sy, Td
Methane oxidation	pmoABC			Ms
Formate oxidation	fdh	As, Ce	Acid, Ce	Ma
Oxygen reduction, Heme Cu Oxidases	cbb3	Sy	Rf, Sk, Sy, Thio	Sy, Td
Oxygen reduction, bd-ubiquinol type	cydA	As, Py, Sy, Tp, Tu	Py, Tp	Thdes
Sulfur reduction	psrA/sreA^c^	Py, Sy, Tu	Py	
Sulfate reduction	dsrAB			Des, Td
Nitric oxide reduction	norB	Py, Tu	Py	
Nitrate reduction	narG		Sk	
**STRESS RESPONSE**				
Arsenite efflux-detoxification	arsB	As, Py, Sy, Tu	Sk, Sy	Sy
Arsenate reduction-detoxification	arsC	Ce, Sy	Ce, Sy	
Mercuric reductase	merA		Thio	
Heavy-metal ATPases	znta		Sy, Thio	Ma, Sy
Superoxide dismutase	sodA	As, Py, Tu	Acid, Py, Thio	Mp
Hydroperoxide reductase (peroxiredoxin)	perox	As, Ce, Sy, Tp, Tu	As, Ce, Sk, Sy, Tp	Ma, Sy, Ta, Td, Thio
Desulfoferrodoxin (superoxide reductase)	sorA	Ce, Tp	Ce, Tp	
Rubredoxin	rub	Ce, Sy	Acid, Ce, Sy	Thdes
Motility	flaB	Sy, Thio	Sy, Thio	Fp, Sy

a Functional genes that code for proteins with high specificity for possible pathway; no genes were found for nitrification (amoA), denitrification (e.g., nirK, nirS, nosZ), methanogenesis (mcrA), thiosulfate oxidase (tqoAB), or arsenite oxidation (aroA = aioA); a ferric reductase from an Acidovorax sp. population was observed in WT.

b Population Types (closest relatives): Acid, Acidovorax sp.; As, Acidilobus saccharvorans; At, Anaerolinea thermophila; Ce, Caldisericum exile; Des, Desulfobacterium sp.; Fp, Fervidobacterium pennivorans; Ma, Methylomicrobium alcaliphilum; Ms, Methylothermus subterraneus; Mp, Mucilaginibacter paludis; Py, Pyrobaculum sp.; Tu, Thermoproteus uzoniensis; Thio, Thiovirga sulfuryoxidans; Sy, Sulfurihydrogenibium sp.; Sk, Sulfuricurvum kujiense; Tp, Thermofilum pendens; Td, Thiobacillus denitrificans; Ta, Thermocrinis albus; Nl, Nitrosoarchaeum limnia; Thiom, Thiomonas sp.; Thdes, Thermodesulfobacteria.

c includes unclassified DMSO proteins that may be related to sulfur and/or arsenic reduction in the Thermoproteales (Jay et al., 2015).

Metabolic evidence for the fixation of carbon dioxide (CO_2_) via the reductive TCA cycle (e.g., ATP citrate lyase; Takacs-Vesbach et al., 2013) was identified in all streamer communities, and was especially evident in *Sulfurihydrogenibium* (Aquificales) populations (Table 4). Copies of acetyl-CoA carboxylase (*accA*) were noted in several bacterial phylotypes as well as a Thermoproteales population in IP 348S. In bacteria, acetyl-coA carboxylase is required for the synthesis of fatty acids. Consequently, the phylogenetic identity of these genes is essentially consistent with the major bacterial phylotypes present across the 3 streamer communities. In the *Archaea*, acetyl-CoA carboxylase is involved in the 4-hydroxybutyrate/3-hydroxyproprionate CO_2_ fixation cycle (or decarboxylase version) (Berg et al., 2007); however, this gene was only observed in the *Thermofilum pendens*-like population present in one of the IP streamer communities (348S), and other key marker genes for the 4-HB/3-HP pathway were not observed (Berg et al., 2007, 2010). Consequently, the sequence data suggest that the primary mechanism of CO_2_ fixation in these communities occurs via the reductive-TCA cycle (Beh et al., 1993; Hügler et al., 2007), and supports measurements of dark CO_2_ fixation rates obtained in a prior study (Yang et al., 2011).

Genes coding for proteins known to be important in the oxidation of reduced sulfur species were observed in these communities, and were most-closely related to the dominant bacterial populations present including *Sulfurihydrogenibium, Sulfuricurvum, Thiovirga, Thiobacillus*, and *Caldisericum* spp. (Table 4). Specifically, *hdrAB* genes indicative of a S oxidation pathway (Friedrich et al., 2005) were found in *Sulfurihydrogenibium* sequences, as has been observed in terrestrial sites of YNP (Takacs-Vesbach et al., 2013). Several other key marker genes and pathways for S oxidation (*sqr, sox*) were identified as *Sulfuricurvum, Thiobacillus*, and *Thiovirga* spp., as well as *Sulfurihydrogenibium*-like (Table 4).

Group I Ni-Fe hydrogenases, indicative of H_2_ uptake and oxidation (Viginais and Billoud, 2007), were found in *Sulfurihydrogenibium, Thermofilum, Thermoproteus*, and *Thiobacillus*-like assemblies (Table 4). The hydrogenases present in the *Sulfurihydrogenibium*-like sequence assemblies are most closely related to other Aquificales genera, because the only known *Sulfurihydrogenibium* sp. to contain a Group 1 Ni-Fe hydrogenase is *S. azoricus* (Aguiar et al., 2004; Reysenbach et al., 2009). To date, the *Sulfurihydrogenibium*-like populations characterized in terrestrial sites of YNP do not contain Group I Ni-Fe hydrogenases (Inskeep et al., 2010; Hamamura et al., 2013; Takacs-Vesbach et al., 2013). The higher concentrations of H_2_(aq) (>4 μM) at IP vent sites correlates with the presence of hydrogenases in *Sulfurihydrogenibium*-like sequences found in two replicate streamer communities (348S, 359S).

A near-complete methane oxidation pathway (particulate methane monooxygenase subunits ABC) was identified in the streamers from WT (369S) (with the exception of formaldehyde dehydrogenase). The *pmoABC* genes were most closely related to genes from the gamma-proteobacterium *Methylothermus subterraneus* (95% nt identity for *pmoA*) (Tsubota et al., 2005; Hirayama et al., 2011). Bacterial populations (similar to *M. subterraneus and M. thermalis*) were identified as major taxa in WT streamers (pH ~ 6.1) in both 2007 and 2008 (*n* = 3) (Table 2). Moreover, no *pmoABC* genes were identified in other vent sites. The *pmoCAB* operon architecture (Ward et al., 2004) was not recovered from the metagenome assembly, and a definitive pathway of CO_2_ fixation via formaldehyde assimilation could not be determined, as the key gene for the ribulose monophosphate pathway (3-hexulose-6-phosphate synthase) was not identified. To date, *pmoABC* genes have not been observed in metagenomes from numerous terrestrial sites in YNP (Inskeep et al., 2010, 2013a; Swingley et al., 2012). Moreover, methanotrophs and/or methylotrophs have not been observed as dominant population types in terrestrial thermal habitats characterized to date, despite fairly high concentrations of CH_4_(aq) in some locations (e.g., 1–2 μM; Inskeep et al., 2005, 2013a). Concentrations of CH_4_(aq) measured in vent sites at IP, WT, and MB ranged from 5 to 30 μM (Table 1); however, WT (i.e., pH ~ 6; T ~ 60°C, lower sulfide) was the only site to exhibit abundant methanotrophic population(s). The lower pH values and higher sulfide of vents at IP and MB (Table 1) may preclude methanotrophic populations, as these conditions are not optimum for the oxidation of CH_4_ using O_2_ as an electron acceptor (Tsubota et al., 2005).

Oxygen is an important electron acceptor in thermal vent communities of Yellowstone Lake as evinced by the presence of Type C (cbb3) heme Cu oxidases in many of the dominant bacterial population types, including *Sulfurihydrogenibium, Sulfuricurvum, Rhodoferax, Thiomonas*, and *Thiobacillus*-like populations (Table 4). These types of heme Cu oxidases have been shown to exhibit low K_m_ values for O_2_, and are often found in hypoxic environments (García-Horsman et al., 1994; Jünemann, 1997; Borisov et al., 2011). Ubiquinol oxidases (e.g., *cydA*) were also observed in several archaeal populations present in the highly sulfidic sites at IP (e.g., Desulfurococcales and Thermoproteales; Jay et al., 2014, 2015) as well as in the Thermodesulfobacteria at WT. These oxidases are common in hypoxic environments and may function in respiration or as O_2_ scavenging proteins (Borisov et al., 2011).

Other electron acceptors important for specific members of these communities may include elemental S, arsenate, nitrate, and sulfate (Table 4). Novel DMSO molybdopterins (tabulated as *psrA/sreA*) related to *Sulfurihydrogenibium* and Thermoproteales populations in the IP streamers may play a role in the reduction of elemental sulfur and/or arsenate (Jay et al., 2015), and these metabolisms would be expected within the S-rich streamer fabric (Figure S2). The only evidence of dissimilatory nitrate reduction (*narG*) was found in the *Sulfuricurvum* population from IP. The role of *norB* genes present in several Thermoproteales populations is not fully understood (NorB may also exhibit activity as an oxygen reductase), in part because no evidence for a complete denitrification pathway has been documented in this group of organisms (Jay et al., 2015). The only evidence of sulfate reduction (*dsrAB*) was associated with nonthermophilic populations at WT (i.e., *Thiobacillus, Desulfobacteria*).

## Summary

Subaerial thermal vents in Yellowstone Lake make a significant contribution to the total chloride flux from the Yellowstone hot spot, and exhibit high concentrations of electron donors (e.g., H_2_S, H_2_, CH_4_) capable of supporting active thermal microbial communities. The high concentrations of CO_2_, H_2_S, H_2_, and CH_4_ in thermal vents of Yellowstone Lake (Table 1) are approximately an order of magnitude higher than many terrestrial systems of YNP (Inskeep et al., 2005, 2010, 2013a; Spear et al., 2005). Consequently, geochemical attributes of Yellowstone Lake thermal vents make them unique for geomicrobiological investigation.

The streamer communities from IP were comprised primarily (>80–85%) of *Sulfurihydrogenibium* spp., and these habitats appear to be highly similar to those observed in terrestrial sites where these filamentous bacteria grow in turbulent, sulfidic channels ranging from pH = 6–8 and T = 65–85°C (Reysenbach et al., 2005; Fouke, 2011; Takacs-Vesbach et al., 2013). However, Ni-Fe hydrogenases were identified in *Sulfurihydrogenibium* populations from replicate IP vent communities, and these genes have not been found in terrestrial *Sulfurihydrogenibium* assemblies from MHS where the concentrations of H_2_(aq) are an order of magnitude lower than those measured in subaerial thermal vents. Streamer communities in the higher pH (~6.1), lower sulfide (< 10 μM) habitats of WT contained less *Sulfurihydrogenibium*, which is consistent with the distribution of this organism as a function of sulfide and hydrogen.

All deep thermal vents contained high levels of dissolved CH_4_; however, *Methylothermus* populations and associated *pmoABC* genes were found only in vent biomass from WT (pH 6; lower sulfide). Cultivated *Methylothermus* spp. utilize CH_4_ as an energy source under aerobic and/or microaerobic conditions at optimum temperature and pH values ranging from ~55 to 60°C and 6–7, respectively (Tsubota et al., 2005; Hirayama et al., 2011). This is the first CH_4_ oxidation pathway identified from a thermophile in YNP, and correlates with high levels of CH_4_(aq), low sulfide concentrations, circumneutral pH values, and temperatures near 60°C (Table 1). Other microbial populations observed in WT streamers are consistent with the higher pH of these habitats (relative to IP), and included considerably greater numbers of novel organisms more closely related to moderately thermophilic and/or mesophilic Proteobacteria, Thermotoga, Chloroflexi, Bacteroidetes, and other Aquificales (i.e., *Thermocrinis*-like), as well as a small contribution (~10%) from *Archaea*.

Although bacteria were more abundant in subaerial vent samples obtained in this study, thermophilic archaea were also observed and included several novel groups. Members of the Thermoproteales and Desulfurococcales were the most numerous archaea in sulfidic habitats from IP, and their occurrence in elemental S streamers is consistent with observations from other circumneutral geothermal environments distributed globally. In contrast, the deep vents in WT and MB contained greater numbers of Euryarchaeota, Korarchaeota, Aigarchaeota, and Thaumarchaeota. The presence of archaea potentially involved in methanogenesis (e.g., *Methanosarcina, Methanospirillum* spp.) may be supported by the high concentrations of CO_2_ and H_2_ in these vent waters. Members of the Korarchaeota were observed primarily in vents from WT, and represented one of the important archaeal groups amplified from vent biomass in both 2007 and 2008. The distribution of korarchaea has been limited to habitats ranging from ~pH 6 to 8 (Auchtung et al., 2011; Miller-Coleman et al., 2012; Inskeep et al., 2013b); consequently, this may be one factor explaining why members of this phylum were not found in other vent samples. Our results document the presence of novel populations not found hitherto in geothermal habitats of YNP. Moreover, these populations exhibit functional attributes consistent with the geochemistry of thermal vent habitats, such as high concentrations of dissolved CO_2_, H_2_S, H_2_, and/or CH_4_.

## Methods

### Sampling

At least 22 sublacustrine hydrothermal vents were sampled during 2007 and 2008 in the Inflated Plain (IP), Mary Bay (MB), and West Thumb (WT) regions of Yellowstone Lake, Yellowstone National Park (YNP) (Figure 1, Table 1). Lake water from Southeast Arm (SA) was also sampled for comparison to thermal vent samples, because no thermal vents are found in this region of the lake, and this location is nearly 5 km from major vent sites and total heat flux in the northern region. Several prior studies (1996 and 1999) on thermal vents from these locations (Balistrieri et al., 2007) provided important background information regarding the general location and properties of vent fluids. However, given the number of vents within these active regions, coupled with the difficulty of locating and sampling vents, the vents sampled here are not necessarily identical to those sampled in prior studies. Sublacustrine vent fluids and solid phase samples were collected in September 2007 and September 2008 using a remotely operated vehicle (ROV) tethered to the *Cutthroat* (Figure S1). Thermal vent waters were obtained to minimize mixing with lake water by using Norprene™ tubing attached a small-diameter suction arm inserted directly into vent discharge sites after positioning the remotely operated vehicle (ROV, Figure S1). Vent waters were collected using retractable polycarbonate piston syringes (2) onboard the ROV, or by a peristaltic pump located onboard the *Cutthroat*. A thermocouple located on the end of the sampling arm was used to continuously record temperature during sample collection. Vent biomass and associated sediments were obtained using the side-mounted syringes (port and starboard), or a separate sampling can (2008 samples). Video of several sampling sites show visible “shimmering” caused by hot water discharge, as well as associated filamentous streamer communities; Figures S3–S7).

Geochemical analyses were performed immediately on the *Cutthroat* for time-sensitive constituents (e.g., dissolved oxygen, sulfide, pH); other sample types were either preserved and stored for further characterization at a temporary field laboratory established at Lake Village or at Montana State University (Bozeman). Glutaraldehyde (1% final concentration) was used to preserve samples for field-emission scanning electron microscopy (FE-SEM). All molecular samples were frozen on dry ice, then transferred to a −80°C freezer.

### Aqueous geochemistry

Several chemical species were analyzed onboard the *Cutthroat*, and included Fe^*II*^ and Fe^*III*^ (Ferrozine method; To et al., 1999), total dissolved sulfide (DS) (amine sulfuric acid method; APHA, 1998), pH, and dissolved oxygen (Winkler method; APHA, 1998). Aqueous pH values were obtained using a Fisher Accumet AP-71 m and AP-55 probe equipped with temperature compensation. Additional aqueous samples were filtered (0.2 μm) directly into sterile 50 mL Falcon tubes and refrigerated at 4°C. Two samples were preserved with trace metal grade HNO_3_ (1%) and HCl (0.5%) for analysis using inductively coupled plasma (ICP)-optical emission spectrometry (OES) (Perkin Elmer) and ICP-mass spectrometry (MS) (Aligent Model 7500)] for total dissolved elements including Ag, Al, As, Ba, Be, Bi, B, Ca, Cd, Ce, Co, Cr, Cs, Cu, Dy, Er, Eu, Fe, Ga, Gd, Ge, Hf, Ho, In, K, La, Li, Lu, Mg, Mn, Mo, Na, Nb, Nd, Ni, P, Pb, Pr, Re, Rb, Sb, Sc, Se, Si, Sm, Sn, Sr, Ta, Te, Tb, Th, Ti, Tl, Tm, U, V, W, Y, Yb, Zn, and Zr. One unacidified sample was analyzed for predominant inorganic anions (F^−^, Cl^−^, SO${}_{4}^{2-}$, NO${}_{3}^{-}$, S_2_O${}_{3}^{2-}$, AsO${}_{4}^{3-}$) using anion exchange chromatography (Dionex DX 500; AS16-4 mm column), and aqueous NH${}_{4}^{+}$ using the phenolate colorimetric (A_630 nm_) procedure on a flow injection analyzer (APHA, 1998). Dissolved inorganic carbon (DIC) and dissolved organic C (DOC) were determined on separate samples taken in closed headspace, baked (500°C) serum bottles using a Shimatzu Model TOC-VCSH total C analyzer. Aqueous samples were collected using either the ROV syringe or the peristaltic pump mentioned above to pump vent fluids through a 140 mm diameter filter (0.4μm) into closed 160 mL serum bottles. The concentrations of dissolved gases (H_2_, CH_4_, and CO_2_) were determined in the laboratory using headspace gas chromatography with a dual-channel Varian gas chromatograph (Model CP2900) equipped with thermal conductivity detection (Inskeep et al., 2005). Aqueous geochemical modeling was performed using temperature corrected thermodynamic constants (Allison et al., 1991; Inskeep et al., 2005).

### Characterization of vent biomass

Vent biomass samples (streamers and/or sediments) were analyzed using a field emission scanning electron microscope (FE-SEM) coupled with energy dispersive analysis of x-rays (EDAX). Aliquots of glutaraldehyde (1%) stored samples were aseptically transferred to 10 mm diameter (0.2 μm) filters, washed with nano-pure water, and then placed on Al stubs for sputter-coating with Ir. Imaging was performed at low voltage (1 kV) and small working distances (~4 mm), whereas elemental analysis was performed at 15 kV and 15 mm working distance.

### Microbial community analysis: DNA extraction, amplification and sequencing

Microbial mat samples were analyzed to assess the predominant 16S rRNA gene sequences distributed across different thermal vents. Streamer and/or sediment samples collected using the ROV were immediately placed on dry ice, and stored within 24 h at −80°C. Total DNA was extracted from the samples using the FastDNA SPIN Kit for Soil (Q-Biogene, Irvine, CA). The primers used for near full-length amplification of 16S rRNA genes included the *Bacteria*-specific Bac8f (5′-AGAGTTTGATCC TGGCTCAG-3′) and the *Archaea*-specific Arc2f (5′-TTC CGGTTGATCCYGCCGGA-3′) primers, each coupled with universal primer Univ1392r (5′-ACGGGCGGTGTGTAC-3′). Purified PCR products were cloned using the pGEM-T Vector System from Promega Corp. (Madison, WI), and the inserts were sequenced using T7 and SP6 primers (TGEN, Phoenix, AZ). Resultant sequences were edited, and checked for chimeras.

### Metagenome and pyro-tag sequencing

Four biomass/sediment samples from three thermal vent locations (Inflated Plain, West Thumb, Mary Bay) were subjected to random 454 pyrosequencing and pyro-tag analysis of 16S rDNA amplicons obtained using two different primer sets focused on *Bacteria* and *Archaea*. DNA extractions (as described above) were used to provide starting material for random 454 pyrosequencing and amplification steps necessary to generate short-fragment 16S rDNA amplicons for pyro-tag analysis (Clingenpeel et al., 2011; Kan et al., 2011). Four vent samples (348S, 349S, 359S, 369S) received one-half plate of random sequencing (median trimmed read length = 360–400 nucleotides). A split DNA sample of IP 359S was used to generate a paired-end library, which generated a greater number of longer contigs (Table S2). Assemblies of all five random sequence libraries were generated using both Celera (Version 4.0) and Newbler assemblers (Celera assembly parameters: doOverlapTrimming = 0, doFragmentCorrection = 0, globalErrorRate = 12, utgErrorRate = 150, utgBubblePopping = 1, and useBogUnitig = 0). Newbler assemblies resulted in a larger number of contigs (as well as longer) than those generated with Celera, and were used for subsequent phylogenetic and functional analysis.

### Phylogenetic and functional analysis

Phylogenetic analysis of long-fragment 16S rRNA gene sequences (1200–1450 bp) was accomplished using blastn to identify closest neighbors in Genbank, and by construction of phylogenetic trees compared to known reference organisms. Phylogenetic trees of long-fragment 16S sequences from the domain *Archaea* were prepared using neighbor-joining and maximum parsimony methods (1000 bootstraps). Phylogenetic trees of bacterial 16S sequences are not shown, due primarily to the extensive diversity of different phylotypes present in sediment samples, and the predominance of *Sulfurihydrogenibium*-like organisms in “streamer” samples from Inflated Plain (IP), which are ~99% identical (16S rRNA gene) to populations also found in terrestrial sites of YNP (Reysenbach et al., 2005; Fouke, 2011; Takacs-Vesbach et al., 2013). Classification of short-fragment 16S rRNA pyro-tag sequences (n ranged from ~14,000 to 32,000 sequences per site) was performed (March 18, 2013) using the Ribosomal Database Project (RDP) Naïve Bayesian rRNA Classifier (Version 2.5, Bayesian RDP training set 9, May 2012).

Random metagenome sequence reads (~360–400 bp) obtained from four vent communities were classified using blastx and sorted by G + C (%) content. Assembled environmental sequence data was also classified using blastx and screened for specific functional genes corresponding to known pathways in material and energy transfer. Query DNA sequences known to code for proteins important in the oxidation of reduced chemical constituents or the reduction of a terminal acceptor were used to search (WU-tblastn) the assembled metagenome sequence (gene list identified in Inskeep et al., 2010, 2013a). Environmental sequence fragments exhibiting homology (*E* < 10^−10^) to query sequences were then reanalyzed using NCBI-blastp against the nr database. Positive functional gene hits were considered when (i) the gene fragment length relative to query length was >0.5, (ii) the phylogenetic identity was confirmed to match the primary population types observed using other sequencing protocols, and (iii) the genes have been described previously in similar phylotypes with closed genomes. There is no guarantee that all functional genes are ancestral; however, our criteria reports only those genes which are on contigs with phylogenetic consistency. Two ‘streamer’ samples from Inflated Plain (348S, 359S) yielded significant consensus sequence of the predominant *Sulfurihydrogenibium* populations present; this phylotype(s) represented >70% of the sequence reads in these samples and generated significant assembled sequence (~25 × coverage). Two of the four vent biomass samples did not produce sufficient assemblies to generate adequate coverage of all major phylotypes (especially Mary Bay sediments).

### Sequence data

Individual sequence reads and assembled contigs from four random metagenome datasets of vent communities (348S and 359S from IP; 349S from MB; 369S from WT) are available under the National Center for Biotechnology Information (NCBI) BioProject PRJNA60433. Long-fragment 16S rRNA gene sequences are deposited with GenBank (NCBI) under Accession Numbers KT453543 – KT453636 (file SUB1068923).

### Conflict of interest statement

The authors declare that the research was conducted in the absence of any commercial or financial relationships that could be construed as a potential conflict of interest.

## Supplementary material

Table S1 **Concentrations of all major geochemical constituents measured in sublacustrine thermal vent waters (and lake water from the Southeast Arm) obtained from Yellowstone Lake during September 2007 and 2008**.

Table S2 **Number of sequence reads, read lengths, and assembly (Newbler) statistics of random shotgun sequencing performed on four thermal vent biomass samples collected in 2008**.

Table S3 **Summary of 16S rRNA gene sequences observed in assembled metagenome sequence data from four sublacustrine thermal vent samples in Yellowstone Lake**. The two samples from Inflated Plain were sampled approximately 300 m apart and exhibited highly similar geochemical signatures (Table 1).

Figure S1 **The remotely operated vehicle (ROV) deployed from the *Cutthroat***.

Figure S2 **Biomass and elemental sulfur collected from a thermal vent streamer community at Inflated Plain (359S) using the ROV-mounted sampling chamber (September 2008)**.

Figure S3–S7 **Video clips of several thermal vent sites sampled in Yellowstone Lake (2007-08) using a remotely operated vehicle (ROV)**. Includes 6 files (^*^.wmv): Figure S3 = IP 329 9_9 _2007; Figure S4 = IP 348 9_11_2008; Figure S5 = WT OV 333 9_12_2007; Figure S6 = WT Deep 339 9_18_2007; Figure S7 = MB 349 9_12_2008.
