# Supplementary material for: Geomicrobiology of sublacustrine thermal vents in Yellowstone Lake: geochemical controls on microbial community structure and function
Source: Front Microbiol. 2015 Oct 26;6:1044. doi: 10.3389/fmicb.2015.01044 (PMC4620420; doi:10.3389/fmicb.2015.01044)
Supplement: Table S3 — Summary of 16S rRNA gene sequences observed in assembled metagenome sequence data from four sublacustrine thermal vent samples in Yellowstone Lake. The two samples from Inflated Plain were sampled approximately 300 m apart and exhibited highly similar geochemical signatures (Table 1). [file Table3.DOC]

**Table S3.** Summary of 16S rRNA gene sequences observed in assembled metagenome sequence data from four sublacustrine thermal vent samples in Yellowstone Lake. The two samples from *Inflated Plain* were sampled approximately 300 m apart and exhibited highly similar geochemical signatures (Table 1).

| **Location**  **Year (Site #)** | **NCBI Closest**  **Cultivated Relative^1^** | **Length (bp)** | **Identity**  **(%)** | **Score** | **NCBI No.** | **Contig ID** |
| --- | --- | --- | --- | --- | --- | --- |
| **Inflated Plain 2008 (348S)** | *Sulfurihydrogenibium* sp. YO3AOP1 ***Thermofilum pendens* Hvv3** *Sulfuricurvum kujiense* DSM 16994 *Thiovirga sulfuroxydans* SO07 ***Staphylothermus hellenicus* DSM 12710** ***Desulfurococcus kamchatkensis* 1221n**  ***Acidilobus aceticus* 1904**  ***Thermoproteus tenax***  ***Pyrobaculum calidifontis* JCM 11548** | 1511  1507  962  789  727  605  586  549  346 | 99.0  97.3  97.9  97.7  95.0  94.7  92.7  99.3  97.7 | 1467  1384  903  736  621  508  464  537  322 | NR_074557.1  NR_029214.1  NR_074398.1  NR_040986.1  NR_074532.1  NR_074374.1  NR_041774.1  NR_044683.1  NR_074360.1 | 18987  00230  13620  11663  02323  03515  03683  12059  20699 |
| **Inflated Plain 2008 (359S)** | *Sulfuricurvum kujiense* DSM 16994 *Sulfurihydrogenibium* sp. YO3AOP1 *Thiovirga sulfuroxydans* SO07  *Paludibacter propionicigenes* WB4 *Curvibacter delicatus* 146  ***Thermofilum pendens* Hrk 5**  *Fibrobacter succinogenes* S85  *Ramlibacter tataouinensis* TTB310 *Thioalkalibacter halophilus* ALCO1 *Sideroxydans lithotrophicus* ES-1 | 1413  1236  1172  656  644  635  409  389  355  352 | 97.5  99.5  99.5  92.2  95.0  95.9  87.3  99.5  89.6  96.6 | 1310  1218  1124  498  548  557  236  380  242  317 | NR_074398.1  NR_074557.1  NR_040986.1  NR_074577.1  NR_028713.1  NR_074406.1  NR_074293.1  NR_074643.1  NR_044406.1  NR_074731.1 | 06785  09439  14934  15529  22487  22049  00081  22711  38343  17900 |
| **West Thumb 2008 (369S)** | *Sulfurihydrogenibium* sp. YO3AOP1  *Acidovorax caeni*  *Duganella violaceinigra* YIM 31327  *Methylothermus thermalis* MYH  *Gemmatimonas aurantiaca* T-27  *Bellilinea caldifistulae*  *Sulfurihydrogenibium* sp. YO3AOP1  *Desulfatibacillum alkenivorans* AK-01 *Melioribacter roseus* P3M  *Melioribacter roseus* P3M  *Anaerolinea thermophila* UNI-1  *Dechloromonas agitata* CKB  *Dechloromonas aromatica* RCB | 989  973  847  733  671  581  503  498  498  475  463  360  289 | 99.6  96.3  96.0  95.1  86.3  90.4  96.2  84.3  92.6  88.3  91.0  98.9  98.3 | 977  865  747  619  355  412  441  261  387  305  335  343  270 | NR_074557.1  NR_042427.1  NR_025770.1  NR_043209.1  NR_074708.1  NR_041354.1  NR_074557.1  NR_074962.1  NR_074796.1  NR_074796.1  NR_074383.1  NR_024884.1  NR_074748.1 | 06993  05974  08731  06891  00760  05243  05676  00717  02752  00825  01739  08628  06073 |
| **Mary Bay 2008 (349S)** | *Prolixibacter bellariivorans* F2  *Solirubrobacter soli* Gsoil  *Capnocytophaga canimorsus* Cc5  *Synechocystis* sp*.* PCC 6803  *Geothrix fermentans* H5 | 400  329  322  253  237 | 92.1  89.5  90.8  90.2  95.7 | 306  216  233  172  204 | NR_043273.1  NR_041365.1  NR_074409.1  NR_074311.1  NR_036779.1 | 00439  01962  00432  00450  01921 |

^1^ Although closest cultivated relatives are listed here, these specific ‘*genus species’* must not be over-interpreted as strict phylogenetic assignments (archaeal sequences in bold; see Figure 3 for phylogenetic tree).
