# Supplementary figures and images for: Geomicrobiology of sublacustrine thermal vents in Yellowstone Lake: geochemical controls on microbial community structure and function
Source: Front Microbiol. 2015 Oct 26;6:1044. doi: 10.3389/fmicb.2015.01044 (PMC4620420; doi:10.3389/fmicb.2015.01044)

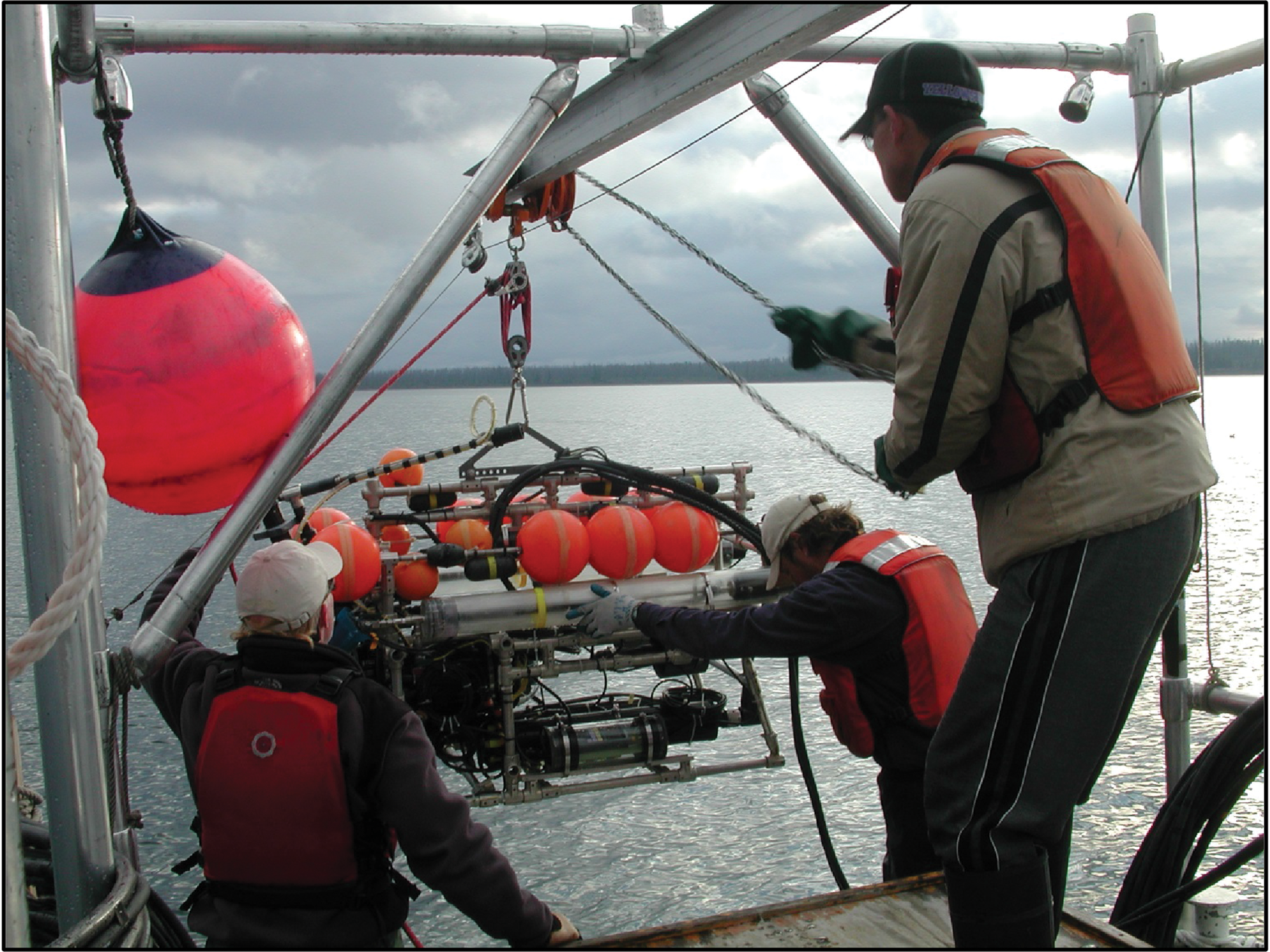

Supplement: Figure S1 — The remotely operated vehicle (ROV) deployed from the Cutthroat. [file Image1.TIF]

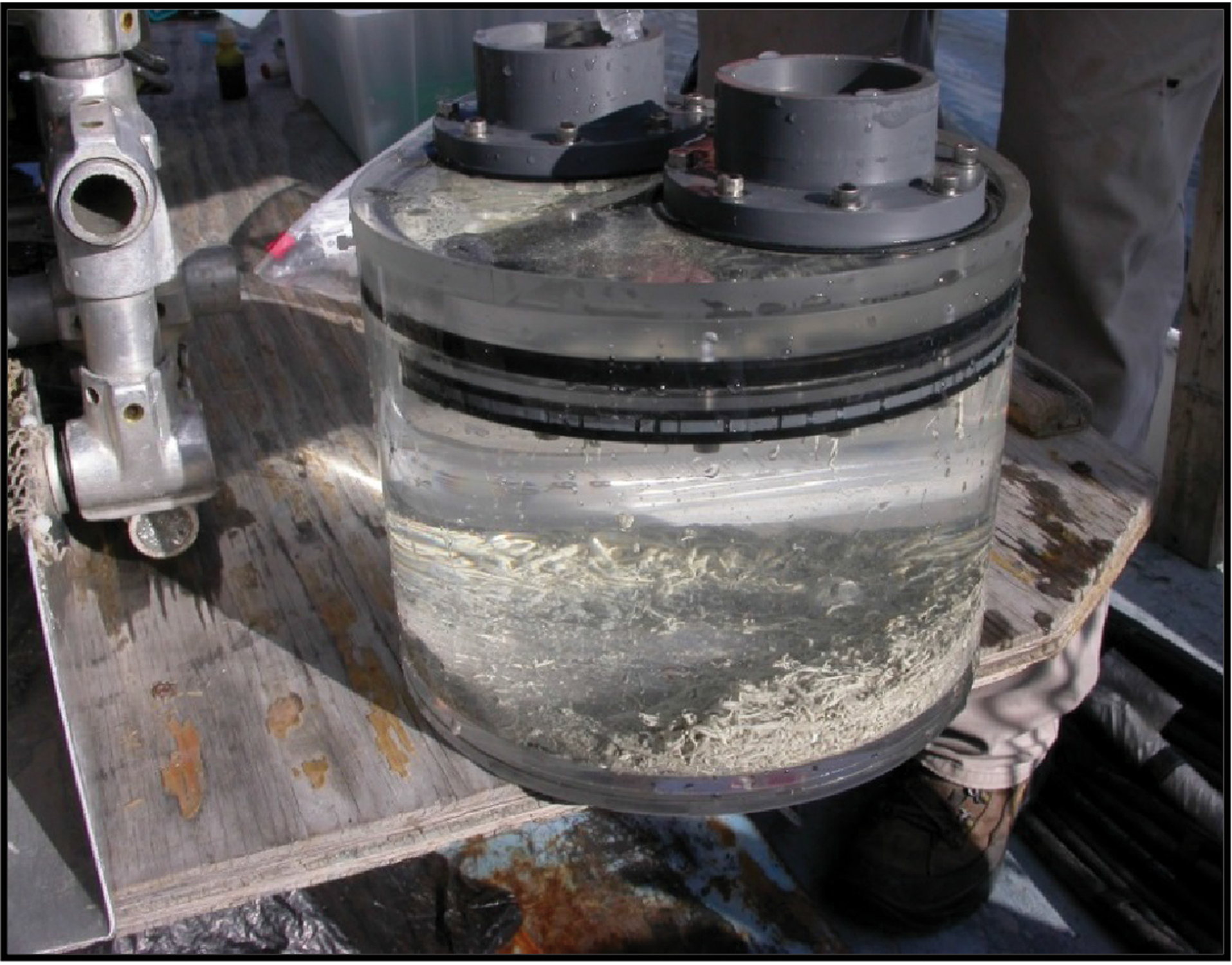

Supplement: Figure S2 — Biomass and elemental sulfur collected from a thermal vent streamer community at Inflated Plain (359S) using the ROV-mounted sampling chamber (September 2008). [file Image2.TIF]
